# A Material Conferring Hemocompatibility

**DOI:** 10.1038/srep26848

**Published:** 2016-06-06

**Authors:** William Everett, David J Scurr, Anna Rammou, Arnold Darbyshire, George Hamilton, Achala de Mel

**Affiliations:** 1 Centre for Nanotechnology & Regenerative Medicine, University College London, London, UK; 2 Interface and Surface Analysis Centre, Boots Science Building, University of Nottingham, University Park, Nottingham, UK; 3 Royal Free Hampstead NHS Trust Hospital, London, UK

## Abstract

There is a need for biomimetic materials for use in blood-contacting devices. Blood contacting surfaces maintain their patency through physico-chemical properties of a functional endothelium. A poly(carbonate-urea) urethane (PCU) is used as a base material to examine the feasibility of L-Arginine methyl ester (L-AME) functionalized material for use in implants and coatings. The study hypothesizes that L-AME, incorporated into PCU, functions as a bioactive porogen, releasing upon contact with blood to interact with endothelial nitric oxide synthase (eNOS) present in blood. Endothelial progenitor cells (EPC) were successfully cultured on L-AME functionalized material, indicating that L-AME -increases cell viability. L-AME functionalized material potentially has broad applications in blood-contacting medical devices, as well as various other applications requiring endogenous up-regulation of nitric oxide, such as wound healing. This study presents an *in-vitro* investigation to demonstrate the novel anti-thrombogenic properties of L-AME, when in solution and when present within a polyurethane-based polymer.

There is a need for biomimetic materials, which can resist thrombosis associated with blood contacting devices which include^1^; cardiovascular implants, such as vascular bypass grafts, synthetic heart valves, vascular access devices, cardiac patches, coatings for stents, pacemakers as well as implants for wound healing. Several materials currently in clinical use such as polytetrafluoroethylene (ePTFE), polyethylene terephthalate (Dacron®) and polyurethane are suboptimal^2^, and often lead to graft failure due to their inherent thrombogenic nature^3^. Ideal materials should possess suitable mechanical properties that provide the required structural support, and mimic the properties of the endothelium; the inner-most blood-contacting tissue in the cardiovascular system. The endothelium, maintains smooth blood flow and nitric oxide (NO) is a key player. NO has the ability to inhibit platelet aggregation and activation, and produce smooth muscle relaxation through activation of the cyclic guanylate monophosphate (cGMP) pathway^4^. It also inhibits smooth muscle cell proliferation. Moreover, NO aids endothelialization, wound healing; although the mechanism in unclear, it has been shown to promote re-epithelialization and collagen formation, as well as angiogenesis^5^.

L-Arginine (L-Arg), a commonly occurring amino acid, is an endogenous substrate of nitric oxide synthase (NOS)^6^, a family of enzymes including neuronal NOS (nNOS), endothelial NOS (eNOS), and inducible NOS (iNOS), which all catalyze the formation of NO (along with citrulline) from L-Arg^7^. L-Arg therefore possesses anti-thrombogenic properties. eNOS produces the majority of the NO within the vascular system, and is present in red blood cells, which means that NO can be produced even in the absence of an endothelium^8^,^9^. Hence, L-Arg is important in cardiovascular physiology, and L-Arg supplementation in individuals with hypercholesterolemia or atherosclerosis has been shown to improve endothelial function, dependent upon L-Arg concentration and the type of cardiovascular disease^10^ There are many analogues of L-Arg that also affect the activity of NOS; L-arginine methyl ester (L-AME) has a very similar chemical structure to L-Arg (Fig. 1a). Although L-AME has relatively low iNOS activity (compared to L-Arg), there have been several studies where L-AME is reported as a substrate for NOS^11^,^12^,^13^,^14^.

L-AME is highly soluble in water, and crystalizes when precipitated from solution. It can therefore be used as an effective porogen when incorporated, in dissolved form, into the bulk of a polymer; following crystallization within the polymer, L-AME can be removed, leaving behind a porous matrix. If left within the polymer, the resulting material would possess the hydrophilic properties of L-AME. Porosity is a highly favorable property for surgical implants, as it facilitates cell-material interactions as well as angiogenesis through the movement of molecules and nutrients within the polymeric scaffold^15^,^16^.

Elastomers are widely used for surgical implants due to their inherent mechanical properties^17^,^18^, which can be adjusted for a range of applications, as well as their ability to act as a host to cell-integrating molecules that can confer biocompatibility when applied as an implant.

In this study, a poly(carbonate-urea) urethane (PCU) is used as a base material and carrier to examine the feasibility of producing an L-AME functionalized material for use in implants and coatings^19^,^20^. It was therefore hypothesized that L-AME, incorporated into PCU, would function as a bioactive porogen, releasing upon contact with blood to interact with eNOS present in blood cells/platelets, resulting in an anti-thrombogenic effect. Base material, carrying the bioactive molecule was characterized for the homogenous presence of the L-AME and its physico-chemical properties, before and after the elution of the bioactive molecule. Whole blood kinetics of L-AME were determined, and L-AME-PCU (L-AME-P) was examined *in-vitro* for its cell-material interaction potential, for blood-contacting surgical implants.

## Results

### Biofunctionalization of the polyurethane based polymer with L-AME

Materials fabricated (as demonstrated in Fig. 2) were tested for successful encapsulation, and homogenous distribution of L-AME within the base material, with both qualitative and quantitative tests.

**Figure 1:**
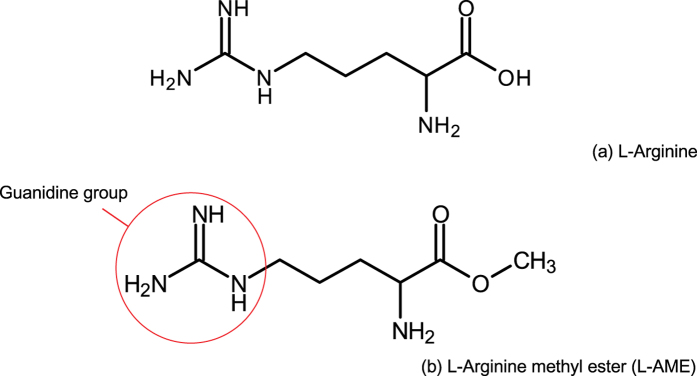
The chemical structures of L-arginine (L-Arg) and L-arginine methyl ester (L-AME). The guanidine group, present in both structures, has been highlighted on L-AME.

The Kaiser test was used to qualitatively observe the presence of primary amine groups through a distinct color change in the presence of L-AME relative to the PCU control^21^. The guanidine group, present on L-AME is identical to that present in L-Arg (Fig. 1). As the functional group is required for L-Arg to be active through the NOS pathway, theoretically its presence allows L-AME to possess the same characteristic^7^,^11^,^12^,^13^,^14^. Fig. 3a(i) shows the unmodified PCU control, in the presence of the reagents, with a non-significant color change, relative to the polymer in the absence of the reagents. Fig. 3b(ii) shows 0.40 M L-AME + PCU, with a distinct overall color change, indicating the presence of L-AME. Furthermore 0.75 M L-AME-P (Fig. 3a(iii)) and 1.05 M L-AME-P (Fig. 3a(iv)) each displayed a distinct color change to deep purple (almost black), relative to the control, indicating the presence of L-AME.

The presence of the amine groups was further confirmed through FTIR. Figure 3b shows the superimposed (matched y-axis magnitude) IR spectrum for a range of L-AME concentrations incorporated into PCU, and suggests the presence of L-AME with an N-H stretch (3500 − 3300 cm^−1^) and an N-H bend (1650 − 1580 cm^−1^) indicating the presence of primary amine groups, with a concentration dependent change in the absorbance at these wavelengths, where an increase in L-AME concentration resulted in an increase in relative absorbance.

ToF-SIMS analysis of the polymer combined with XPS analysis confirmed that L-AME was homogenously distributed within the polymer matrix, with a positive correlation between the concentration of L-AME used and the intensity of the observed peaks. Several of those found in the positive spectrum were the ions at m/z 100, 101 and 127. The ion at m/z 189 was used as a marker for L-AME due to its high intensity. Images were constructed for the peak at m/z 189 (Fig. 3c). XPS data showed a positive correlation between the N+ peak at 401.6 eV of the N 1s signal with the concentration of L-AME-P. The peak at 401.6 eV corresponds to positively charged nitrogen (NH_3_^+^). The positively charged nitrogen, arising from L-AME, rises with the concentration of incorporated L-AME. The respective number of N atoms in the base polymer matrix and L-AME, allowing determination of the L-arginine-polymer ratio N_R_/N_Pol_, where a positive correlation was observed, between the L-arginine-polymer ratio N_R_/N_Pol_ and the concentration of incorporated L-AME.

L-AME, incorporated through physical entrapment, was expected to be contained within the carrier polymer via electrostatic attraction. Figure 4b shows 0.40 M L-AME-P, which has a continuous surface, with the presence of L-AME in cavities under the surface of the polymer. Figure 4d, with 1.05 M L-AME-P has a distinctly different surface from the other L-AME concentrations; (Fig. 4b,c) with protruding areas, roughly hexagonal in shape, and around 2 μm in diameter.

Contact angle measurements using the static sessile drop method demonstrated that these functionalized polymers were hydrophilic in the presence of L-AME. The difference in mean contact angle for each L-AME concentration incorporated within the base polymer was significantly different (p < 0.001) from each other concentrations, ranging from 101.3° for the control to 70.1° for the highest concentration of incorporated L-AME. Figure 5 shows a concentration dependent, inversely proportional, trend in the mean contact angle for increasing concentrations of L-AME, causing an increase in hydrophilicity, supported by linear regression analysis (r^2^ = 0.958, p < 0.001). Following L-AME elution, the material reverted to becoming hydrophobic, with no significant association between initially incorporated L-AME concentration and surface wettability. (r2 = 0.037, p < 0.295).

The total amount of L-AME contained within a test scaffold of L-AME-P was found by calculation to be 1.87 mg (0.40 M), 3.73 mg (0.75 M), and 5.60 mg (1.05 M). OPA assay was used to determine the amount of L-AME eluting from the polymer. The 0.4 M scaffolds eluted at an initial rate of 59.4 (±1.7) μg min^−1^ (after 10 minutes) before the rate became immeasurable. The 0.75 M discs eluted at an initial rate of 143.6 (±2.4) μg min^−1^ (after 10 minutes), and then dropped to a rate of 11.2 (±1.0) μg min^−1^ after 20 minutes before the rate became immeasurable. The 1.05 M scaffolds, (which was the highest concentration) eluted at an initial rate of 230.5 (±2.0) μg min^−1^, and had dropped to 0.14 (±0.01) μg min^−1^ after 42.67 hours.

Figure 4e is the scanning electron microscopy of PCU control, which was treated in the same conditions as to elute L-AME from L-AME-P. The surface of this control remained uninterrupted, with POSS crystals present on the surface-a component of the polyurethane composite used in this study. 0.40 M L-AME-P sample after complete elution of L-AME (Fig. 4f) had a surface covered with shallow pores. The average pore diameter for the 0.40 M L-AME structure was 1.59 μm (±0.33 μm), and the pore density was 1.00 × 10^8^ mm^−2^. 0.75 M L-AME-P (Fig. 4g) developed a deeper porous structure, with an average pore diameter of 2.74 μm (±0.58 μm), and a pore density of 6.98 × 10^7^ mm^−2^. 1.05 M L-AME-P also developed a honeycomb-like porous structure, with an average pore diameter of 2.95 μm (±0.83 μm), and a pore density of 6.01 × 10^7^ mm^−2^ (Fig. 4h). The porous structure was most likely produced by re-crystallization of L-AME within the polymer substance, following the evaporation of the solvent during the polymer casting process. There was a significant correlation between the L-AME concentration and pore size (R^2^ = 0.494, p < 0.001); as the incorporated concentration of L-AME was increased, the pore size increased, which is different to the most common occurrence with porogen, where the pore size is determined by the size of the porogen molecule^22^. With L-AME-P, the pore density significantly decreased with an increase in incorporated L-AME (R^2^ = 0.844, p < 0.001).

The control had a higher maximum stress of 66.4 MPa and a similar percentage elongation at break of 740.4%. In general, an increase in incorporated L-AME caused a decrease in mechanical strength, with the lowest maximum strength (42.6 ± 3.79 MPa) of 1.05 M L-AME and strain at break (634.6 ± 86.3%) of 0.75 M L-AME. The elution of L-AME caused a decrease in the modulus of the polymer, with the lowest maximum strength (34.3 ± 4.85 MPa) and strain at break (555.6 ± 55.1%) for 1.05 M L-AME.

### Cell-material interactions of the bioactive scaffolds

#### Whole Blood Kinetics with Thromboelastography

The control, (in the absence of L-AME), demonstrated the lowest time until clot formation initiated (SP) at 8.38 mins, until the clot reached 2 mm in amplitude (R) at 9.75 mins, and until the clot reached 20 mm in amplitude (K) at 3.45 mins. The control also had the highest angle at 48.78^o^, with maximum amplitude of clot formation at 57.38 mm, and G of 7027 d/sc. 191 mM L-AME solution had the highest SP time, R, and K at 17.42 mins, 20.33 mins, and 7.52 mins respectively. 191 mM L-AME solution also had the lowest angle at 27.78^o^, maximum amplitude of 47.70 mm, and clot strength (G) of 4612 d/sc.

There were found to be significant differences across all measured variables, with the results summarized in Table 1. L-AME, a recognized analogue of L-Arg, has been reported as a NOS substrate^11^,^23^,^24^. This was confirmed by our group, by performing TEG tests with relevant L-AME samples with the blood of eNOS knockout mice, kindly provided to us by Prof Adrian Hobbs’s group (QMUL) as a collaboration study. The unpublished results, which led us to the current study examining L-AME, demonstrated that the concentration-dependent anti-thrombogenic effect was not evident in the presence of eNOS knockout blood and the TEG parameters had no significant differences across the varied concentrations.

L-AME-P samples were found to have significant differences across all measured variables, summarized in Table 2.

The control polyurethane had the lowest SP time, R and K at 9.30 mins, 10.63 mins and 2.97 mins respectively. The control also had the highest angle at 51.57^o^, with maximum amplitude of 59.67 mm and G of 7397 d/sc. 1.05 M L-AME-P had the highest SP time, R, and K at 21.47 mins, 25.10 mins and 7.03 mins respectively. 1.05 M L-AME-P also had the lowest angle at 30.07^o^, maximum amplitude of 52.50 mm, and a clot strength of 5538 d/sc.

#### Cell-Material Interactions on L-AME Functionalized Scaffolds

Endothelial progenitor cells (EPC), which were isolated from blood, and cultured for 10 days on scaffolds of increasing L-AME concentrations, indicated that the presence of L-AME significantly increases cell viability (Fig. 6a). On the fifth day, at the higher concentrations of L-AME (0.75 M, 1.05 M ), L-AME significantly improved cell viability when compared with both the control and washed samples, showing that the honeycomb structure of the polymer has a less significant role for this particular cell-type. At day 10, cell-viability followed a significant L-AME concentration-dependent response. Furthermore, the highest 1.05 M L-AME concentration significantly increased cell-metabolic activity at a higher rate compared to the eluted sample (1.05 M eluted) and the control (SI Fig. 1). Scaffold porosity appeared to significantly improve cell-viability at 1.05 M and 0.40 M L-AME. Immunohistochemistry images (Fig. 6b) support the cell viability data and the characteristic EPC colonies are prominent and much more dense in the highest L-AME concentrations.

Fibroblasts were cultured on L-AME-P scaffolds for 72 hours to investigate the potential for application in wound-healing devices, while endothelial progenitor cells were used to investigate the potential for application in a vascular device. Results (Fig. 6c) from this cell-type indicate that the lowest concentration, 0.40 M L-AME, is optimal for fibroblast viability, showing a significant difference at 72 hours. In both time-points tested, scaffold porosity seemed to significantly increase cell-viability compared to the untreated scaffold. Furthermore, immunohistochemistry images (Fig. 6d) support the Alamar Blue assay data and show favorable interaction with the porous scaffold.

## Discussion

This study, for the first time, has demonstrated the anti-thrombogenic properties of an L-arginine analogue, L-AME, the potential for its incorporation into a polyurethane based polymeric carrier, and its influence as a porogen on the carrier material for cell material interactions. The presence of L-AME was confirmed by identifying the primary amine group through distinct color changes, with increasing L-AME concentrations. Further quantitative confirmation was carried out using FTIR, that showed a dependent change in the absorbance at the demonstrated wavelengths, where an increase in L-AME concentration resulted in an increase in relative absorbance. Moreover, L-AME proved to be homogenously distributed within the polymer matrix, with a positive correlation between the concentration of L-AME used and the intensity of the observed peaks.

Hydrophilicity is an important feature for synthetic vasculature, as it can prevent platelet adhesion^25^,^26^, and so alter fibrinogen adsorption, and promote endothelialization, which are all crucial for long-term graft patency^15^,^27^,^28^,^29^. A prompt release response within L-AME-P in the presence of a hydrophilic environment is highly favorable as the patency of blood contacting implants are influenced by the proteins encountered immediately after implantation, governed by the Vroman effect^19^,^23^. Following L-AME elution, the material reverted to its original state of surface wettability thus suggesting that the increase in hydrophilicity was due to the presence of an increasing concentration of L-AME. However, it is recognized that the determination of hydrophobicity of porous structures through contact angle measurements is less reliable as the measurements are influenced by the surface property of the porous structure with the capillary forces as well as roughness and microstructures within pores. However comparison with a single porogen as used here reduces this variability. Thromboelastography studies performed on L-AME eluted, porous scaffolds demonstrated no difference when compared with unmodified polyurethane, suggesting that the anti-thrombognic properties observed are due to the presence of L-AME, but homogenous pore structures, which are less than 5 μm diameter (Fig. 4) induced by L-AME elution had negligible influence in thrombogenicity.

A controlled, steady, and sustained release of L-AME is desirable, preferably at a rate that enables the biological processes to support graft patency through biomimicry of a functional blood-contacting tissue. For further study, an area of interest would be to examine the modification of L-AME release simultaneously with other known anti-thrombogenic molecules that influence the levels of NO, or endothelialization for further applications^30^,^31^. Further work should also examine techniques such as the production of multi-layered polymers, or microencapsulation of molecules, to facilitate prolonged, sustained release of L-AME^32^.

Porosity is also an important topographic feature for cell-material interactions within a scaffold^15^. In the lowest concentration of L-AME-P, the pores were distinct, but separate. In the polymer with the highest concentration of incorporated L-AME however, the structure showed interconnection between the pores. The even distribution of pores across the surface produced a honeycomb-like structure, as the pore distribution and inter-pore distance is potentially as important as pore size^20^,^22^,^33^,^34^,^35^ proved to be ideal for cell seeding. The concentration-dependent changes of porosity observed here presents the possibility of producing custom scaffolds, of desired pore dimensions, for a given application.

Favorable mechanical properties are one of the advantages of using polyurethane based materials for surgical implants^33^. The mechanical properties of the polyurethane base used in this study, were comparable to those from a previous study^19^,^36^. Following L-AME incorporation, a decrease in strength noted in comparison with the control, but yet it is still more than sufficient to withstand the mechanical stresses that the material would be subjected to in an *in vivo* environment^15^.

The experimental procedures currently undertaken to determine blood compatibility of biomaterials are somewhat controversial and is a subject of discussion amongst researcher as well as policy makers^37^,^38^.

Traditionally, standardized blood compatibility tests are classified into five categories: thrombosis, blood coagulation, platelets function, hematology and complement system^39^.

Thromboelastography is a more complete method of coagulation testing than other plasma-based alternatives^40^,^41^, and proved the potent anti-thrombogenic properties of L-AME^42^. Thromboelastography by using whole blood incorporates all cellular elements involved in the hemostatic process, including platelets, red cells and white blood cells thereby giving a more global representation of the coagulation process as it occurs *in vivo*. In detail, the results demonstrated, that the effect is primarily on the plasmatic component (SP and R – Table 3) with less activation of coagulation at the higher concentrations used. There are also significant effects observed with K and alpha angle (Table 3), which are related to the rate, amount and speed of thrombin generation, and in the case of alpha angle, also the rate at which fibrin crosslinking occurs. The significant differences indicate that thrombin generation is significantly attenuated and thus the material is less thrombogenic. The relatively small impact on the MA (function of fibrinogen and platelets), indicated that it is likely that there is no significant reduction (or increase) in platelet reactivity.

L-AME maintained its anti-thrombogenicity when incorporated into the base material, PCU, which is itself marked improved upon the hemocompatibility of currently available synthetic materials^43^. Improved hemocompatibility is of paramount importance for maintaining graft patency^3^,^44^.

With favorable hemocompatibility and cell-material interaction, implants based on L-AME-P could potentially have successful long-term integration. NOS is weakly stimulated by its substrate under conditions of relative substrate abundance, and therefore the ability of L-AME is expected to be relatively greater. It would be of interest, in subsequent studies, to look into the detailed pathways of L-AME-initiated anti-thrombogenicity, as well as its antiplatelet synergy with others such as clopidogrel and prostacyclin, as well as direct NO donors including nitrosothiols and diazeniumdiolates^44^,^45^,^46^.

The long-term patency of blood contacting surgical implants is preserved in the presence of a functional endothelium. *In-situ* endothelialization is considered the most favorable option, where endothelial progenitor cells (EPC) can be exploited^29^. In the current study, the EPC viability was higher than the control even with the lowest concentration of L-AME samples. Furthermore, significantly high cell viability with L-AME eluted scaffolds suggest a role for topographical features (Fig. 4) on the EPC cell behavior as material topography is well recognized to influence cell-material interactions. Thus both the L-AME released from the scaffolds and the porous structure of L-AME-P might have a synergistic effect.

Materials for wound healing implants also require favorable blood-material interactions, as well as efficacious interactions with EPC, particularly for angiogenesis and arginine has been previously linked to anti-aging and wound healing^47^,^48^,^49^. It was therefore of interest to investigate the growth of fibroblasts on the scaffolds, which had already proved to be optimal for hemocompatibility, and EPC growth. This effect of positive influence on relatively low concentrations has been observed previously^50^.

The results of this study indicate that L-AME possesses great potential for promoting endothelialization and wound healing. This suggests that L-AME has a dual effect, by first initiating favorable metabolic cascades, and second, by creating a uniformly porous surface that improves cell adhesion (as shown in our fibroblast experiments). Our results further support previous evidence that administration of L-Arginine improves wound-healing capacity in mice by activating the NOS pathway and increasing NO- levels^49^,^51^. Further work could potentially include investigating the internalization pattern of L-AME, and protein expression of NOS. In addition, studies could also tests how the surface area of the porous matrix/limiting dimensions of porosity that could influence the platelet/fibrinogen adsorption and the protein excretion of the cells, which might be a potential issue for a long-term vascular implant, in addition to *in-vivo* investigation of L-AME incorporated material in the form of appropriate surgical implants

In conclusion, the results of this study support the hypothesis that L-AME, incorporated into polyurethane-based polymer, elutes into a hydrophilic environment, to exhibit an anti-thrombogenic effect. In addition, L-AME-incorporated material has demonstrated optimal cell adhesion properties, to support long-term cell-materials interactions, for use in surgical implants. As an anti-thrombogenic agent, L-AME has broad application in the cardiovascular field, with the potential to increase hemocompatibility for synthetic vascular grafts, coated stents, pacemakers, blood gas sensors, and many other blood-contacting medical devices. If L-AME is active through NOS, the determination of which must be a priority in further study, it has potential for application in wound healing, as well as many other applications that require endogenous up-regulation of NO levels.

## Methods

All the chemicals used in this study were obtained from Sigma-Aldrich Co. LLC.

### Producing a polyurethane-based polymer with incorporated L-AME (L-AME-P)

**Figure 2:**
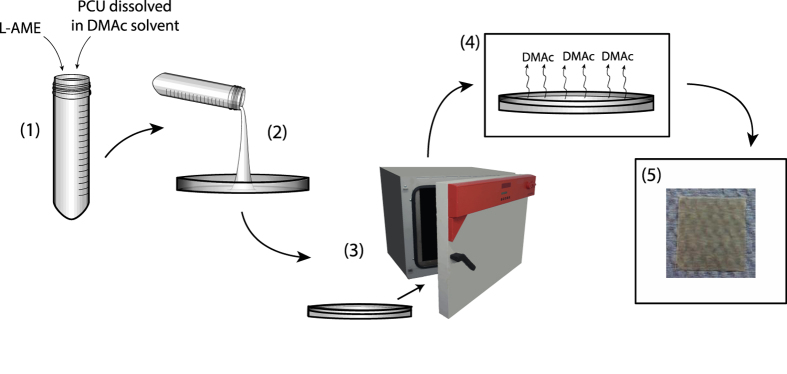
L-AME-P synthesis: Physical entrapment method. *Cast method* (1) L-AME combined with PCU (dissolved in DMAc solvent) (2) L-AME-P slurry poured into glass container (3a) Container placed into oven at 60 °C for 24 hours (4a) DMAc evaporates from solution (5a) L-AME-P produced.

**Figure 3:**
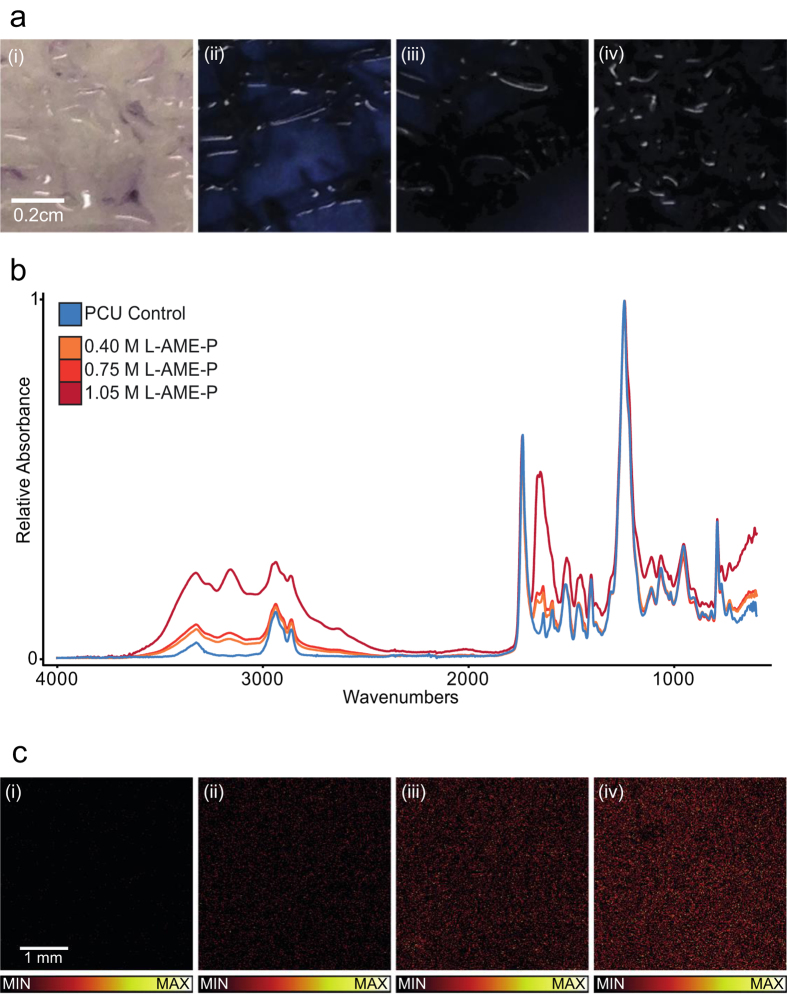
(**a**) Kaiser test on polymer; coloration indicated the presence of primary amine groups, and so confirms the presence of L-AME: (i) POSS-PCU control – A hint of purple coloration (ii) 0.40 M L-AME-P – Distinct overall color change to purple (iii) 0.75 M L-AME-P – Overall color change to deeper purple (iv) 1.05 M L-AME-P – Distinct overall color change to deep purple/black, corresponding to the higher concentration of L-AME. (**b**) Superimposed (matched y-axis magnitude) FTIR spectra for POSS-PCU with increasing concentrations of incorporated L-AME: POSS-PCU control and 0.40 M/0.75 M/1.05 M L-AME + POSS-PCU. The spectra produced suggest the presence of L-AME with an N-H stretch (3500 − 3300 cm^−1^) and an N-H bend (1650–1580 cm^−1^) indicating the presence of primary amine groups, with a dependent change in the absorbance at these wavelengths, where an increase in L-AME concentration resulted in an increase in relative absorbance. (**c**) ToF-SIMS analysis of the polymer combined with XPS analysis. Images were constructed for the positive ion at m/z 189. The images show a uniform distribution of the L-AME as well as an increase in ion intensity with the concentration used in the preparation of the surfaces: (i) Positive ion image of m/z 189 (C_7_H_17_N_4_O_2_^+^) for the POSS-PCU control. The scale is offset so all images have the same scale (MC = 5). (ii) Positive ion image of m/z 189 (C_7_H_17_N_4_O_2_^+^) for 0.40 M L-AME-P. The scale is offset so all images have the same scale (MC = 5). (iii) Positive ion image of m/z 189 (C_7_H_17_N_4_O_2_^+^) for 0.75 M L-AME-P. (iv) Positive ion image of m/z 189 (C_7_H_17_N_4_O_2_^+^) for 1.05 M L-AME-P.

**Figure 4:**
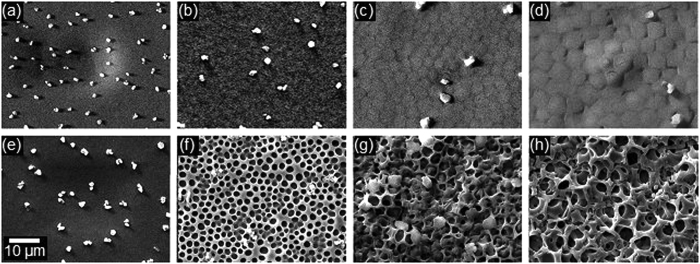
Surface of PCU with increasing concentration of L-AME incorporated without elution, magnified at 5000x: (**a**) PCU control (**b**) 0.40 M L-AME-P (**c**) 0.75 M L-AME-P (**d**) 1.05 M L-AME-P. Surface of PCU following elution of L-AME from the polymer matrix, magnified at 5000x: (**e**) PCU control (**f**) 0.40 M L-AME-P (**g**) 0.75 M L-AME-P (**h**) 1.05 M L-AME-P. Incorporation, and subsequent elution, of L-AME produced a porous structure, with an increase in initially incorporated L-AME causing an increase in pore size, with the higher concentrations developing a honeycomb-like porous structure. POSS nanoparticles are present on the surface of all samples of polymer as the polyurethane used in the study is a nanocomposite of PCU.

**Figure 5:**
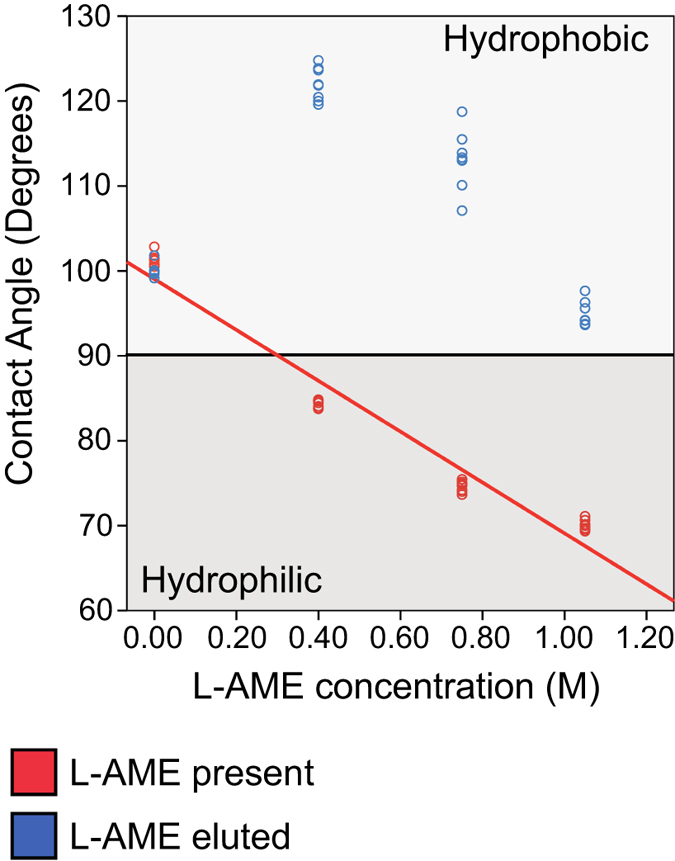
Mean Contact Angle (Theta) of water on the surface of L-AME-P with increasing concentration of incorporated L-AME, ranging from the PCU control to 1.05 M L-AME. L-AME present: An increase in incorporated L-AME caused a significant decrease in the surface contact angle, supported by linear regression analysis (R2 = 0.958, p < 0.001). L-AME eluted material: No statistical relation between initially incorporated L-AME concentration and surface contact angle (p = 0.295).

**Figure 6:**
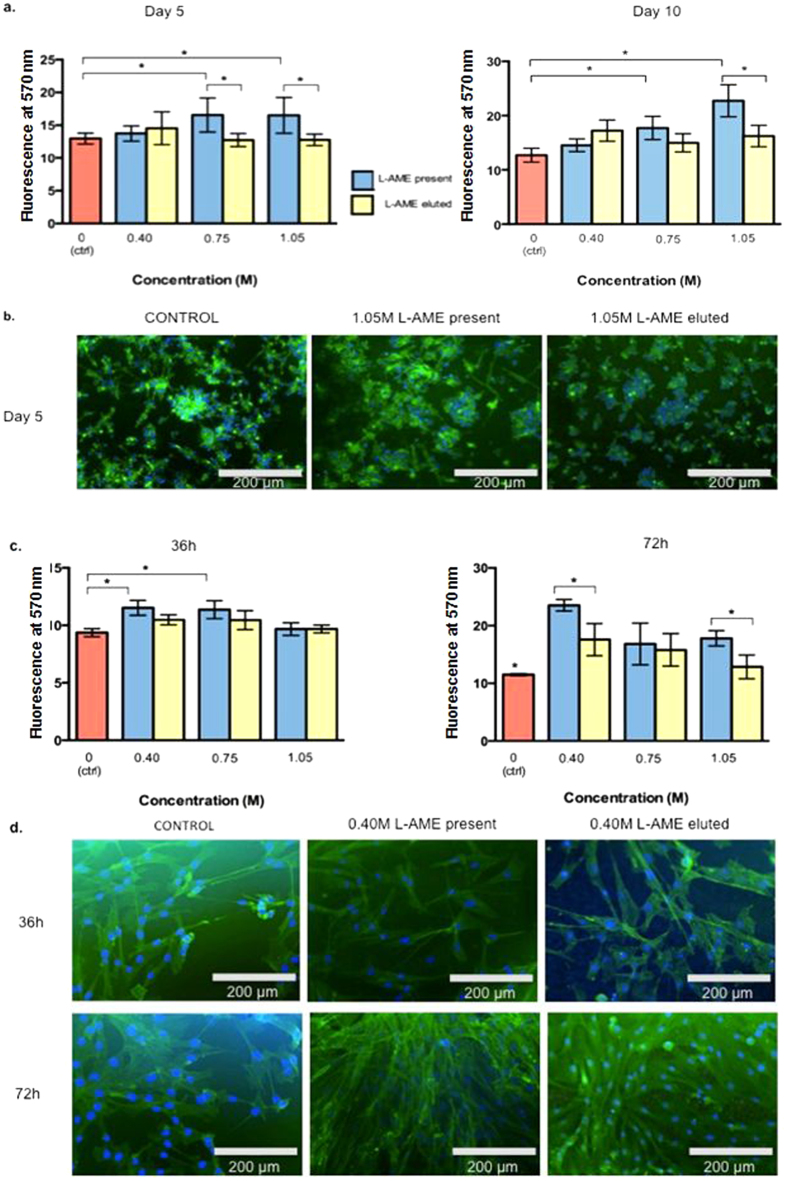
(**a**) EPC viability analysis (O.D values ± SD) by Alamar Blue at day 5 and day 10. (**b**) Immunohistochemistry images at day 5 for 1.05 M L-AME present and washed. Cell nuclei were stained with DAPI (blue) and actin was stained by phalloidin (green). (**c**) Fibroblast viability analysis (O.D values ± SD) by Alamar Blue at 36 h and 72 h. (**d**) Immunohistochemistry images at 36 h and 72 h for 0.40 M L-AME present and washed. Cell nuclei were stained with DAPI (blue) and actin was stained by phalloidin (green). *p < 0.05 Bonferroni shows statistical significance.

L-arginine methyl ester dihydrochloride (L-AME.2HCl) was dissolved in water (500 μL) at various concentrations (0–540 mg mL^−1^), and dimethylacetamide (DMAc) (5 g) was then added. Each resulting L-AME.2HCl solution was added to 18%(w/w) PCU polymer (0.9 g PCU in 4.1 g DMAc). PCU synthesis has been reported previously^36^. The resulting mixture was poured into a circular glass tray (radius = 46 mm), and heated at 60 ^o^C in a circulating oven for 12 hours to facilitate DMAc evaporation (Fig. 2).

### Biofunctionalization of the polyurethane-based polymer with L-AME

#### O-phthalaldehyde (OPA) assay for the determination of L-AME concentration in solution

Fluorescent products are formed through the reaction of a primary amine with o-phthalaldehyde^52^,^53^. Based upon a previously used method, 83 μL of 2-mercaptoethanol solution (25 μL made up to 4 mL using ethanol) and 883 μL borate buffer (0.05 mol L^−1^, titrated to pH 9.0) were mixed with 34 μL of analyte solution. After standing for 2 hours, 34 μL OPA solution (10 mg made up to 1 mL using ethanol) was added to the mixture and the fluorescence was recorded within four minutes following mixing. Fluorescence was determined using a Fluoroskan Ascent FL fluorescence plate reader with a 355 nm excitation filter and a 460 nm emission filter. Values were obtained using a standard curve for absorbance against concentration (r^2^ = 0.948, the standard curve can be found in the Supporting Information, SI Fig. 2).

#### L-AME elution from L-AME-P

Discs of 0.40 M/0.75 M/1.05 M L-AME-P with a radius of 7.8 mm produced using a Trotec 100R laser cutter were placed in a container with 4.0 mL of 0.9% NaCl, and were placed onto a roller at 37 °C. Eluents were changed periodically, whilst re-incubating the samples in new containers with 0.9% NaCl. OPA assay was performed on the removed eluents to test for the presence of L-AME.

#### Tensile strength of L-AME-P

Samples of polymer were tested to determine the mechanical strength of the polymer with increasing concentration of incorporated L-AME (untreated group). It was also determined whether elution of incorporated L-AME affected the mechanical properties of the polymer (partial elution group). The tensile strength of L-AME + PCU was tested using an Intron 5500 series (5565) Twin Column material testing system and Bluehill^®^ software. The polymer was cut to specification ISO 37 Type 3, and the Instron grips were set at a distance of 20 mm. Half of the dumbbells from each L-AME concentration were treated with H_2_O for seven days to allow complete elution of L-AME from the polymer.

#### Contact angle analysis of L-AME-P surface

Polymer cast onto glass slides was placed into a Kruss DSA 100 to measure contact angles using the static sessile drop method. 5 μL of H_2_O (Ström) was dropped at a rate of 60 μL min^−1^ onto L-AME-P, and the contact angles were measured using Drop Shape Analysis for Windows 1.92.1.1 exactly 20 seconds after the droplet came into contact with the polymer surface. Two samples were used, with four separate measurements taken per sample.

#### Fourier transform infrared spectrometry (FTIR) of L-AME-P polymer surface

L-AME-P polymer prepared on glass slides was analyzed using a JASCO FT/IR-420 spectrometer. 25 scans were performed across the range 4000 − 600 cm^−1^ at a resolution of 4 cm^−1^. Analysis was performed using Essential FTIR 3.10 build 41.

#### Kaiser test for the presence of L-AME on polymer surface

This qualitative test is based upon the reaction of ninhydrin with primary amine groups^21^. 5% (w/v) ninhydrin solution (1 g made up to 20 mL with ethanol), phenol 4:1 (w/v) in ethanol, and 2% (v/v) potassium cyanide (0.4 mL of a 1 mmol L^−1^ aqueous solution of potassium cyanide was made up to 20 mL with pyridine) were produced. 100 μL of ninhydrin solution was added to the surface of the L-AME + PCU polymer, followed immediately by 50 μL of phenol solution, and then 50 μL of potassium cyanide solution. Each treated surface was then photographed using the same settings and external conditions.

#### Scanning electron microscopy of L-AME-P

Polymer for imaging was either untreated, or washed with de-ionized H_2_O for seven days to allow complete elution of L-AME. A 3 × 3 mm^2^ section of polymer was placed onto a stub. The stubs were inserted into a Quorum Q150 Rotary-Pumped Sputter Coater, which coated the samples with gold. The stubs were then placed in a Zeiss Evo HD15 SEM, and the samples were examined.

#### Time-of-flight Secondary Ion Mass Spectrometry (ToF-SIMS) analysis of L-AME-P

ToF-SIMS uses an ion beam to determine the mass of particles on the surface of a solid material^54^. Samples of polyurethane-based polymer with increasing concentration of incorporated L-AME were cast onto glass slides for surface analysis. The TOF-SIMS analyses were executed using an ION-TOF TOF-SIMS IV instrument (Munster, Germany), equipped with a Bi LMIG. Stage scans with 256 pixels mm^−1^ and 5 frames per patch (1 shot per pixel) allowed to analyze larger zones of 4000 × 4000 μm^2^. The mass resolution (FWHM) at m/z 15 was on average 3368 ± 83 (n = 7). The positive ion mass spectra were calibrated with m/z 1 (H^+^), 15 (CH_3_^+^), 29 (C_2_H_5_^+^), 43 (C_3_H_7_^+^) and 57 (C_4_H_9_^+^).

#### X-ray photoelectron spectroscopy (XPS) analysis of L-AME-P

XPS spectra were acquired on a Kratos Axis Ultra (Kratos, UK) with a monochromated Al kα source (1486.7 eV) using an emission current of 10 mA and an anode potential of 12 kV. Wide scans were acquired with a pass energy of 80 eV; high resolution scans were acquired using a pass energy of 20 eV. Charge neutralization was used. A wide scan and a high resolution C 1 s, O 1 s, N 1 s and Si 2p scan of three different spots on each sample were acquired. The samples were cut into 1 × 1 cm^2^ pieces using a diamond glasscutter as to fit the XPS sample holder. The XPS spectra were analyzed using CasaXPS software (version 2.3.16).

### Whole-blood kinetics with thromboelastography.

#### Thromboelastography (TEG) on a series of L-AME solutions

Solutions from 0–191 mM L-AME concentration (in water) were produced. Blood was taken from healthy volunteers, after signing an informed consent document accordance with the regulations and as approved by the local (Royal Free Hospital campus) ethics committee. Blood was collected in 2.7 mL 3.0% sodium citrate BD Vacutainers®. 20 μL of L-AME solution and 20 μL of 0.2 M CaCl solution were added to a TEG cup with 320 μL of citrated blood and TEG measurements (Table 3) were obtained using a TEG® 5000 Thromboelastograph® Hemostasis Analyzer System, and analyzed as described by Wohlauer *et al*.^40^, producing a visible tracing (SI Fig. 3).

#### TEG with L-AME-P polymer

Discs of polymer with a radius of 7.8 mm, were placed into the wells of a 24-well plate, and 500 μL of citrated blood (obtained as described in section 2.4.2) was then pipetted into each well. The plate was then incubated at 37 ^o^C for 1 hour on a PSU-10i orbital shaker at 50 RPM. After incubation, 340 μL of blood was removed from each well, and added to a TEG cup that contained 20 μL of 0.2 M CaCl solution. Thromboelastography was performed, and the resultant traces were extracted.

### Cell-material interactions on L-AME functionalized scaffolds

Details of EPC extraction have been reported previously^30^,^55^. Whole blood was obtained from healthy volunteers after signing an informed consent document accordance with the regulations and as approved by the local (Royal Free Hospital campus) ethics committee. Approximately 40 ml of whole human blood was placed in 2.7 ml light blue-capped Vacutainer® citrate tubes (BD USA). 3 ml of Histopaque was added to six 15 ml centrifuge tubes, and 3 ml of whole blood was layered onto the Histopaque. The material at the opaque interface was transferred into two clean centrifuge tubes. 20 ml of HBSS (Life Technologies) was added, mixed gently and centrifuged at 250 g for 10 minutes. The supernatant was discarded and the pellet re-suspended in 5 ml HBSS and mixed gently. This centrifugation and supernatant-discarding process was repeated twice more. The cells were then re-suspended in 5 ml cell culture medium M199 (Life Technologies) with 10% FBS (Life Technologies), and 1% penicillin and streptavidin (Life Technologies). Cells were counted using Trypan blue exclusion dye, using 10 μl Trypan blue, and 10 μl cell suspension. Cells were stored in a flask at 37 °C with 5% CO_2_ for three days. Cells were seeded at a density of 35 × 10^3^cells cm^−2^ in a 24-well plate.. Culture medium was replenished every 3 days, and cells were examined under light microscopy every 3 days. AlamarBlue® assay (Life Technologies) was performed according to standard protocol at day 5 and day 10.

Fibroblasts were grown at 37 °C with 5% CO_2_ for four days.. Cells were seeded at a density of 25 × 10^3^cells cm^−2^ in a 24-well plate. Culture medium was replenished every 36 h, and cells were examined under light microscopy every 24 h. AlamarBlue® assay (Life Technologies) was performed according to standard protocol at 36 h and 72 h and excitation and emission was recorded at 360 nm and 460 nm respectively. Cells on polymer were fixed in 4% formaldehyde stained with phalloidin (actin stain) overnight and DAPI (4′,6-diamidino-2-phenylindole) (DNA/nucleus stain) for 10 min according to standard protocol with manufacturer-supplied instructions.

### Statistics

IBM^©^ SPSS^©^ Statistics (Version 22) software was used to conduct analysis of variance, one-way ANOVA, Kruskal-Wallis and linear regression analysis.

## Additional Information

**How to cite this article**: Everett, W. *et al*. A Material Conferring Hemocompatibility. *Sci. Rep.* **6**, 26848; doi: 10.1038/srep26848 (2016).

## Supplementary Material

Supplementary Information

## Figures and Tables

**Table 1:** Summary of thromboelastography results for increasing concentrations of L-AME in solution.

L-AME Concentration (mM)	SP (min) One-way ANOVA p < 0.001	R (min) One-way ANOVA p < 0.001	K (min) One-way ANOVA p < 0.001	Angle (deg) One-way ANOVA p < 0.001	MA (mm) One-way ANOVA p = 0.045	G (d/sc) One-way ANOVA p = 0.031
0 (Control)	8.38	9.75	3.45	48.78	57.38	7027
19	9.85	11.33	3.52	47.18	56.03	6505
38	11.43	12.75	4.13	44.68	54.67	6194
57	12.22	14.15	4.72	39.18	51.18	5350
77	12.48	14.37	5.23	36.75	51.43	5333
115	13.08*	15.33*	5.43	35.18	50.32	5123
153	16.48***	19.05***	6.72**	30.55**	49.48	4984(a)
191	17.42***	20.33***	7.52***	27.78***	47.70	4612(a)

n = 6. One-way ANOVA showed a significant difference between concentrations for each variable. Bonferroni post-hoc test showed significant differences for concentrations relative to the control.

*p < 0.05 **p < 0.005 ***p < 0.001 ^(a)^n = 5 due to incomplete Thromboelastography tests.

**Table 2:** Summary of Thromboelastography results for increasing concentrations of L-AME incorporated into POSS-PCU polymer.

L-AME Concentration (M)	SP (min) Kruskal-Wallis p = 0.01	R (min) Kruskal-Wallis p = 0.005	K (min) Kruskal-Wallis p = 0.012	Angle (deg) One-way ANOVA p < 0.001	MA (mm) One-way ANOVA p = 0.002	G (d/sc) One-way ANOVA p = 0.002
0.00 (Control)	9.3	10.63	2.97	51.57	59.67	7397
0.4	11.73	13.2	3.67	45.83	55.7	6287
0.75	15.53	18.23	5.07	36.03*	54.87	6081*
1.05	21.47*	25.10*	7.03	30.07**	52.50**	5538**

n = 3. Kruskal-Wallis Test and one-way ANOVA showed a significant difference between concentrations for each variable. Pairwise comparisons (Kruskal-Wallis) and Bonferroni post-hoc (after ANOVA) test showed significant differences for concentrations relative to the control.

*p < 0.05 **p < 0.005 ***p < 0.001.

**Table 3:** Variables measured from a thromboelastography tracing^36^.

Parameter	Abbreviation	Measurement	Interpretation
Split Point [mins]	SP	Time until earliest detected resistance	Time to until initial clot formation
Reaction time [mins]	R	Time until 2 mm amplitude	Enzymatic clotting factor initiation phase
Coagulation time [mins]	K	Time from 2 mm–20 mm amplitude	Rate at which clot strengthens, representative of fibrinogen to fibrin cleavage
Angle [degrees]	α	Angle of tangent to the line between R and K	
Maximum amplitude [mm]	MA	Maximum recorded amplitude of tracing	End result of platelet-fibrin interaction
Clot strength [d/sc]	G	Calculated from amplitude of tracing	Total clot strength
