# Supplementary material for: A Material Conferring Hemocompatibility
Source: Sci Rep. 2016 Jun 6;6:26848. doi: 10.1038/srep26848 (PMC4893622; doi:10.1038/srep26848)
Supplement: Supplementary Information [file srep26848-s1.pdf]

Supporting Information

A Material Conferring Hemocompatibility;

Applications in Blood-Contacting Surgical Implants

William Everett[1], David J Scurr[2],Anna Rammou[1],

Arnold Darbyshire[1], George Hamilton [1,3],

Achala de Mel[1*]

[1]Centre for Nanotechnology & Regenerative Medicine, University College London, London, UK

[2]Interface and Surface Analysis Centre, Boots Science Building, University of Nottingham, University Park, Nottingham, UK

[3]Royal Free Hampstead NHS Trust Hospital, London, UK

*Dr Achala de Mel

Lecturer in Regenerative Medicine

UCL Centre for Nanotechnology & Regenerative Medicine

Division of Surgery & Interventional Science, University College London;

Tel: 020 7794 0500 Ex: 35375

Email: a.mel@ucl.ac.uk

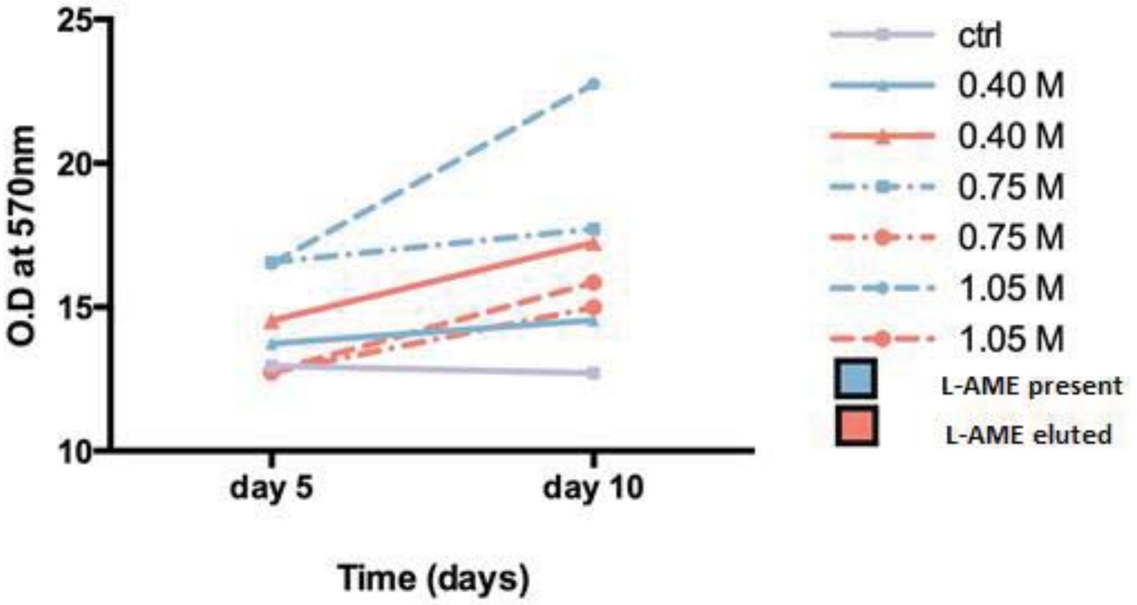

SI Figure 1
EPC viability shown as optical density over time. Blue lines indicate L-AME present samples. Orange lines indicate L-AME eluted samples. The 1.05M with L-AME present shows a significantly higher rate of increase in the cell viability ($p<0.05$, Bonferroni)

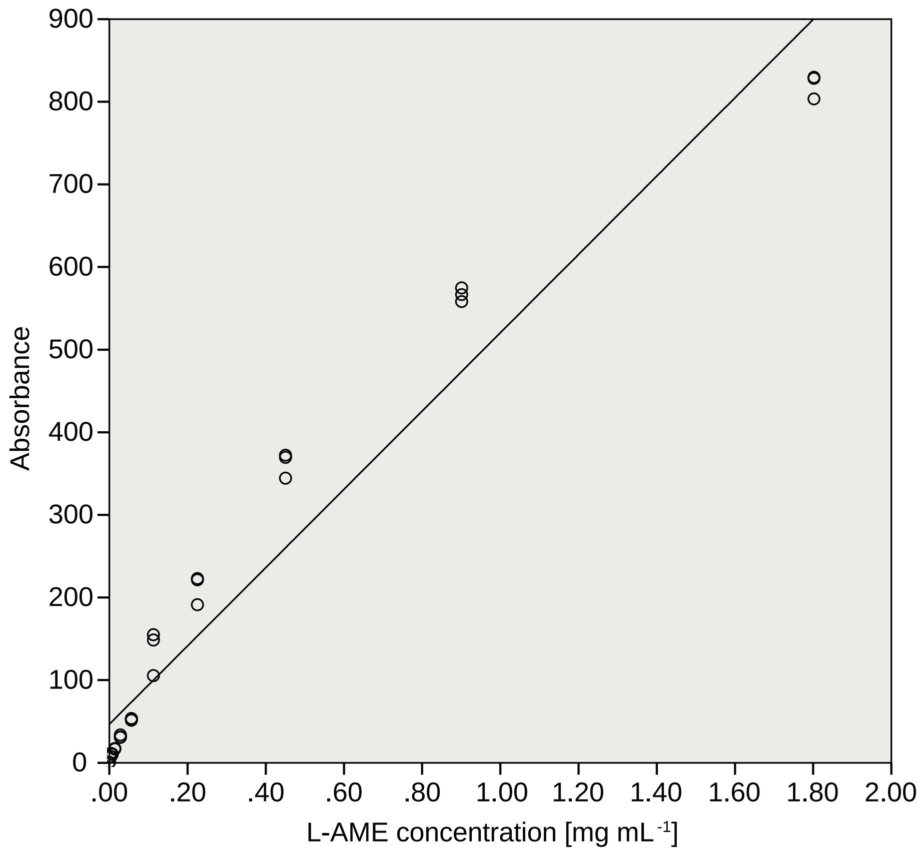

SI Figure 2

Standard curve for o-phthalaldehyde (OPA) assay for the determination of L-arginine methyl ester (L-AME) concentration in solution.

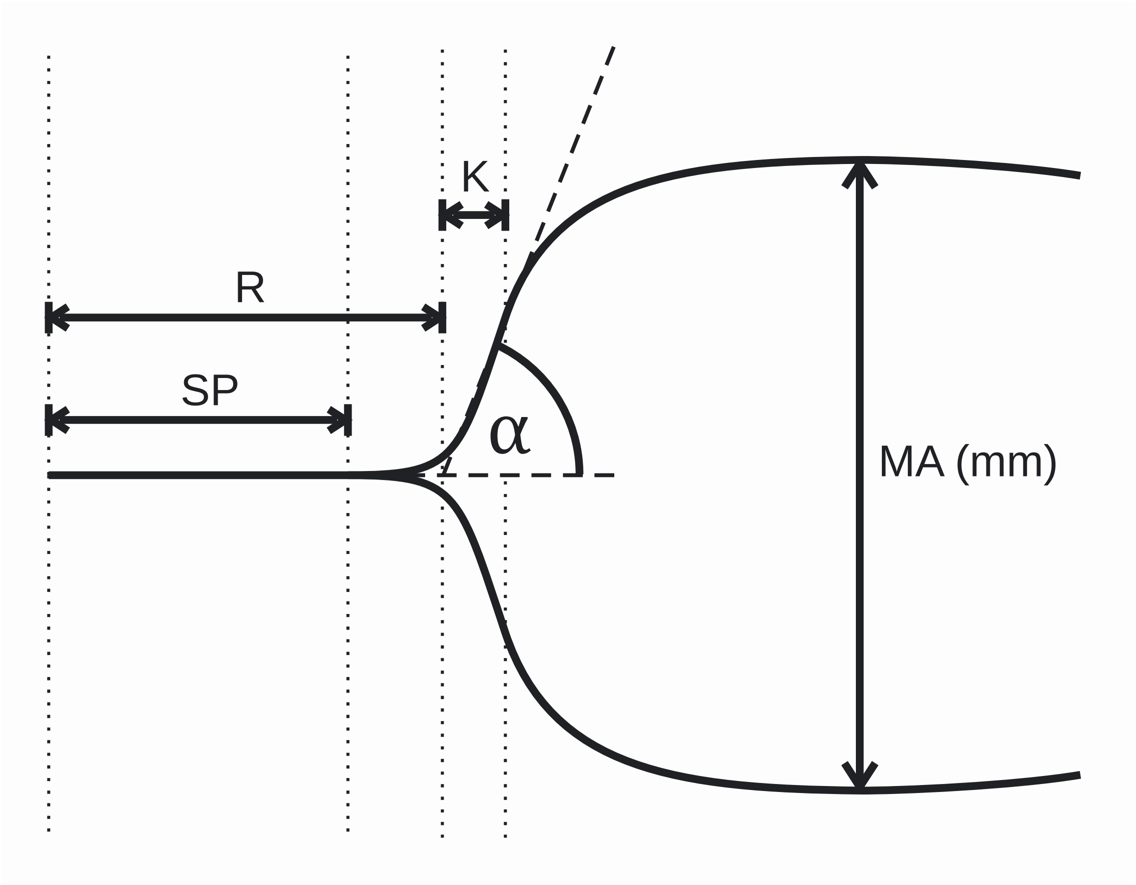

SI Figure 3

Example of a tracing produced by the TEG® 5000 Thromboelastograph® Hemostasis Analyzer System. Variables measured from the tracing: Split point (SP), reaction time (R), coagulation time (K), angle (α), maximum amplitude (MA), clot strength (G).

A Material Conferring Hemocompatibility; Applications in Blood-Contacting Surgical Implants

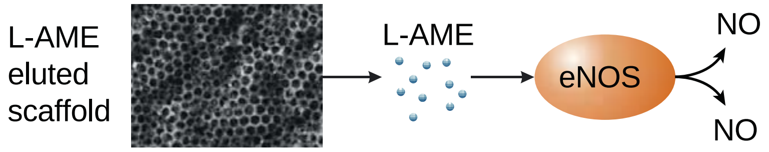

L-AME incorporated within a honeycomb-structured polyurethane-based polymer, elutes upon contact with blood. eNOS present in blood cells / platelets may be potentially producing nitric oxide (NO), resulting in the observed anti-thrombogenic effect.
